# Dewatering Hypersaline Na_2_SO_4_ and NaCl via Commercial Forward Osmosis Module

**DOI:** 10.3390/membranes16010014

**Published:** 2025-12-31

**Authors:** Noel Devaere, Vladimiros G. Papangelakis

**Affiliations:** Department of Chemical Engineering and Applied Chemistry, University of Toronto, 200 College St., Toronto, ON M2S 3E5, Canada; noel.devaere@mail.utoronto.ca

**Keywords:** forward osmosis, hypersaline brine, salt rejection, hollow fibre membrane, reverse salt flux, minimum osmotic differential, polyamide thin-film composite

## Abstract

Efficient water recycling in the hydrometallurgical industry requires the dewatering of hypersaline Na_2_SO_4_ or similar brines via non-evaporative methods. Unfortunately, many non-evaporative methods require the use of specific solutes and are not compatible with complex hydrometallurgical effluents. Forward Osmosis (FO) uses a draw solution to link known non-evaporative water recycling methods with feed solutions that are otherwise incompatible. There is minimal experimental data on the dewatering performance of today’s available commercial FO membranes, especially with hypersaline concentrations (>70,000 mg/L total dissolved solids). This study tests the commercially available Aquaporin HFFO2 hollow fibre FO membrane module with hypersaline Na_2_SO_4_ or NaCl feed solutions versus a MgCl_2_ draw solution. It identifies a key requirement to maintain water flux above a certain threshold to prevent a decrease in Na Rejection or an increase in Mg reverse flux. It also defines a minimum osmotic differential that can be used to parameterize water flux, similar to the temperature of approach in heat exchangers, but to determine the extent of water removal in FO. We demonstrate that even under mildly acidic conditions, existing FO membranes can concentrate Na_2_SO_4_ to saturation, paving the way for their use in the hydrometallurgical industry.

## 1. Introduction

Closing the water balance in hydrometallurgical processes is an important step towards improving the sustainability of metal production [1]. Hydrometallurgical streams and effluents are often highly concentrated inorganic solutions, requiring energy-intensive dewatering processes to recycle and reuse the reclaimed water [2,3]. Additionally, due to the common adoption of H_2_SO_4_ in hydrometallurgical flowsheets, the solutions are predominantly sulphate-based instead of chloride-based and acidic [4]. Additionally, a lot of water capture and recycling test work has been conducted in the literature; this has been mainly on seawater purification for drinking water. In addition, sparingly soluble salts such as gypsum (CaSO_4_·2H_2_O) supersaturate during the water capture and recycling process, fouling heat exchange equipment and Reverse Osmosis (RO) membranes alike [5]. This has sparked interest in developing new technologies for hydrometallurgical water recycling, such as Forward Osmosis (FO) [2,6]. There are limited FO performance data available for hydrometallurgical streams, which are often more concentrated than seawater and sulphate-based [2,7,8,9].

Forward osmosis (FO) is a membrane technology that utilizes an osmotic differential to selectively separate water from a feed solution into a draw solution. There are several recent reviews that summarize and explain how FO works [10,11,12,13,14]. For hydrometallurgical effluents, FO is promising because it allows the drawing of water from a mixed/complex brine into a single-salt draw solution having higher osmotic pressure, spontaneously. The single-salt draw is then dewatered in the form of ice by a freeze concentration (FC) step [6].

This FO–FC process is of particular interest to Na_2_SO_4_ streams, a common hydrometallurgical waste salt resulting from the neutralization of acidic SO_4_ solutions with NaOH. Concentrating Na_2_SO_4_ without evaporation is currently important to improve the efficiency of electrolyzers for acid and base recycling without dramatic increases in capital or operating expenses [15,16]. Our FO–FC process typically uses a MgCl_2_ draw solution rather than another soluble low-cost electrolyte such as NaCl due to its high solubility and low eutectic temperature (−33.6 vs. −21.4 °C for NaCl). The latter allows reaching higher osmotic pressures (401 vs. 311 bar for NaCl as per OLI Studio V12.0). CaCl_2_ could also be used as a draw solute, having a eutectic temperature of −51.8 °C; however, reverse diffusion of Ca^2+^ into SO_4_^2−^ rich feeds would cause gypsum scaling. Due to the above, we chose MgCl_2_ as our default draw solution to investigate. To validate this process concept, FO performance data using a concentrated Na_2_SO_4_ feed and a MgCl_2_ draw solution is needed.

Unfortunately, the literature data are sparse for FO on hypersaline feed solutions (greater than ~70,000 mg/L total dissolved solids). In osmotic terms, hypersaline means feed osmotic pressures beyond 50 bar, the typical osmotic pressure of seawater retentate from a reverse osmosis (RO) process [3,17]. Hypersaline feeds have greater viscosity and density compared with seawater (see Table S1 in the Supplementary Materials). This causes them to have lower diffusivities and experience greater concentration polarization [17]. Additionally, hypersaline feeds require higher draw solution concentrations, which increases the internal concentration polarization of the membrane [18]. In our previous studies [2,6] we used industrial samples, whereas this work investigates feeds with a one-component solution of NaCl and Na_2_SO_4_ at high concentration with an initial 50 bar osmotic pressure to simulate an RO retentate. The goal is to understand and capture the performance metrics of a commercial FO membrane using highly soluble salts that would not produce scaling problems.

Data on the separation performance of commercial FO modules is also limited [12,19,20,21]. Four commercially available FO membranes are summarized in Table 1. Compared to those identified in the recent review by Abounahia et al. [14], only half of the commercial membranes reported remain available. Further, certain formats of membranes are no longer available from their manufacturers. For example, the Aquaporin membranes are no longer manufactured in a flat sheet or 0.6 m^2^ hollow fibre formats. Early characterization was performed with these formats instead of what is available today. Since every membrane has unique properties, it is essential to measure its separation performance against solutes of interest under conditions close to the intended application, as there are yet to emerge guidelines for acceptable performance [22]. In a previous study [2], we used a flat sheet cellulose triacetate (CTA) membrane (Fluid Technology Solutions). In contrast, in this work, we used a polyamide thin-film composite (PA–TFC) membrane, which is expected to be more resilient to acid hydrolysis [23]. The Aquaporin HFFO^®^ 2 membrane used in this study has a thinner active layer (79.8 ± 9.8 nm) [21]; incorporates functional proteins which aid in water-salt selectivity [24,25]; uses a hollow fibre geometry which enables greater membrane area packing density; and is commercially available. For hydrometallurgical streams below pH 3, pH adjustments can be made as reported in our earlier work [2].

In this work, we investigated the separation performance of concentrating hypersaline Na_2_SO_4_ and NaCl solutions with a commercial FO membrane. We quantified the membrane’s rejection of feed contaminants and reverse draw solute flux to identify the extent to which FO can concentrate hypersaline feeds before performance is compromised. Additionally, we introduced and justified the use of a minimum osmotic differential parameter to characterize performance, such as the temperature of approach in a heat exchanger [30]. We also compared membrane performance for feed solutions at acidic pH = 3 and at neutral pH = 7, which fall within the membrane’s operating specifications.

## 2. Materials and Methods

### 2.1. Chemicals and Equipment

Feed and draw solutions were prepared from stock salts of NaCl (99%, Thermo Fisher Scientific, Waltham, MA, USA), Na_2_SO_4_·10H_2_O (99%, Thermo Fisher Scientific, Waltham, MA, USA), and MgCl_2_·6H_2_O (99%, Thermo Fisher Scientific, Waltham, MA, USA). The initial and final concentrations of the solutions are shown in Table 2. FO experiments used 2500 g of feed solution initially at 50 bar osmotic pressure: 5.9 wt% NaCl and 12.9 wt% Na_2_SO_4_. The 50 bar osmotic pressure composition was chosen to represent the retentate from a conventional RO process. The MgCl_2_ draw solution had an initial mass of 2500 g and concentrations of 13.8 wt% for Na_2_SO_4_ and 14.6 wt% for NaCl feeds. The different starting concentrations were required to compensate for the different osmotic pressure versus concentration responses of the two feed solutes and maintain similar osmotic differentials across the experiments. The temperature was 22 ± 1 °C across all solutions and tests.

The pH was adjusted by dropwise addition of 2 N HCl or 0.2 N NaOH for NaCl; 2 N HCl or 30 wt% MgO slurry for MgCl_2_ solutions; and 1 N H_2_SO_4_ or 0.2 N NaOH for Na_2_SO_4_ solutions. The acids and bases were chosen to avoid adding any new ions to the initial solution. Solutions were characterized by inductively coupled plasma optical emission spectroscopy for elemental concentrations (5900 ICP–OES, Agilent Technologies Canada, Mississauga, ON, Canada), pH via electrode (Fisherbrand, 13-620-631), density via hydrometer (Fisherbrand, 11-583D), and water activity via a vapour pressure osmometer (LabMaster-aw Neo, Novasina, Lachen, Switzerland).

The membrane used in this study was a commercial Aquaporin HFFO2 (Aquaporin, Kongens Lyngby, Denmark) [26]. This is a commercial hollow fibre membrane module with 2.3 m^2^ of area. The membrane was run in FO-mode with the feed on the fibre’s lumen side (facing active-layer) and draw on the shell side (facing support-layer). The FO membrane properties were quantified previously with a DI water feed for draw solutes, including NaCl (A = 1.56 L/m^2^/h/bar, B = 0.24 L/m^2^/h, S = 150 μm) and MgCl_2_ (A = 1.86 L/m^2^/h/bar, B = 0.07 L/m^2^/h, S = 160 μm) [21]. A is the water permeability coefficient, B is the salt permeability coefficient, and S is the structural parameter of the support layer.

### 2.2. FO Apparatus

**Figure 1:**
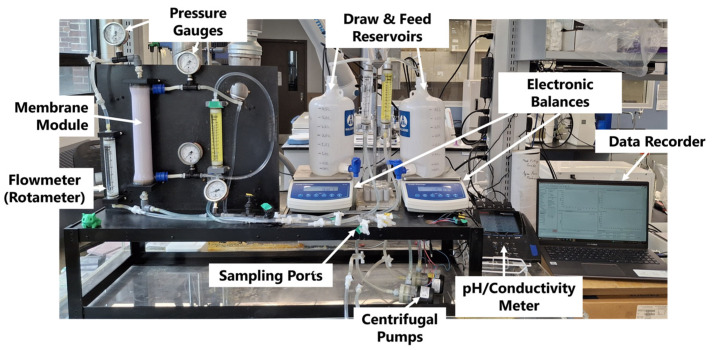
The FO experimental setup.

The FO experiments were conducted using the setup detailed in Figure 1. Two 4 L carboys (Nalgene, Thermo Fisher Scientific, Waltham, MA, USA) served as tanks for the feed and draw solution, each of which rested atop electronic balances (PB-350, Cole-Parmer Canada, Quebec, QC, Canada). The spigots at the bottom of the carboys were plumbed to centrifugal pumps (893-10 24 VDC, March Pump, Glenview, IL, USA) located under the balance, and they circulated the feed and draw solutions through independent loops in the system. The solutions were each pumped through a rotametere (F-400, Blue-White Industries Ltd., Huntington Beach, CA, USA) to measure the flow rate, then into the vertically suspended Aquaporin FO module (HFFO2, Aquaporin, Kongens Lyngby, Denmark). The feed solution passed through the lumen side of the fibres, and the draw solution passed through the shell side of the FO module (also known as FO-mode or Active Layer Feed Side, AL-FS). Pressure gauges monitored the pressure (0.1–0.2 bar-g) at each entrance and exit stream in the FO module to ensure appropriate transmembrane pressure (TMP < 4 bar). Conductivity (013005MD, Thermo Fisher Scientific, Waltham, MA, USA) and pH probes were inserted into each tank to monitor the solution composition throughout the trial. The electronic balances and electrochemistry meter reported their values every 5 s to a custom LabVIEW data acquisition system (DAQ). Lastly, 4 sample valves were located after the pumps and before the discharge for the collection of ICP samples.

### 2.3. FO Procedure

The operating conditions used are summarized in Table 2, and each condition was duplicated for each solute and pH combination. The FO experiments began with the system primed with RO water, while the tanks were loaded with the initial feed and draw solutions. At the start, the tank spigots were opened, the pumps turned on, and one holdup volume of the feed (210 mL) and draw (230 mL) were purged via the discharge tubes. After the purge, the system was allowed to stabilize for 90 s when the first samples were taken from all ports and then again, every 60 s. After the 4th sample, the duration was increased to every 120 s since the water flux was much lower. The system was halted after the 7th and final sample at 700 s. At this endpoint, the osmotic differential was near 0 bar, so the water flux was near 0 LMH. The initial experimental conditions were chosen to be at the same osmotic pressure (rather than concentration) so that the endpoint occurred at the same time for both feeds to maintain consistent sampling. During each experiment, we used the two electronic balances to monitor water flux stability online through our DAQ, which recorded and displayed the water flux within 5 s. Since the mass removed from the feed solution had to be gained by the draw solution, our setup could assess the process mass balance in real time to ensure sampling occurs only under mass-balanced conditions (see Supplementary Materials Figure S3b). Additionally, we sampled all inlets and outlets simultaneously to obtain a pseudo-steady state approximation of the FO membrane’s single-pass performance. The pseudo steady state approximation is useful for performance estimates in process modelling. These estimates are needed to estimate the cost of large-scale and long-duration piloting experiments, which measure membrane stability and fouling.

After the experiment, the system was drained, and both loops were immediately purged with 5 holdup volumes of RO water to remove any remaining salts. The system was then flushed with RO water, after which the tanks were switched out for the next experiment. If there was more than 24 h between experiments, a drop of saturated CuSO_4_ solution was added to the RO water to inhibit the growth of any biofoulants during idle time and protect the membrane.

### 2.4. Calculation and Measurement of FO Operating Metrics

The water flux was calculated by fitting a linear model to a 25 s window of mass-time data recorded by the electronic balances. The slope (g/h) was then converted to a water flux via:

(1) $$J_{W}=\frac{a_{t-25}}{\rho_{H_{2}O}A_{mem}},$$

where *J_W_* is the water flux (L/m^2^h, LMH), *a*_*t*−25_ is the mass vs. time slope over a 25 s window (g/h), *ρ_H_*_2*O*_ is the density of water (997 g/L), and *A_mem_* is the area of the membrane (2.3 m^2^).

The specific reverse draw salt flux ($\frac{J_{s}}{J_{w}},\;mmol/L_{Flux}$) was calculated through a solute balance on the feed side:

(2) $$\frac{J_{s}}{J_{w}}=\frac{V_{Out,F}C_{Out,F}-V_{In,F}C_{In,F}}{A_{mem}J_{W}\;},$$

where *V_Out_*_,*F*_ and *V_In_*_,*F*_ denote the outlet and inlet volumetric flow rate (L/h) of feed solution, respectively, and *C_Out_*_,*F*_ and *C_In_*_,*F*_ are the outlet and inlet concentrations (mmol/L) of the draw cation in the feed solution, respectively.

The membrane rejection (*R*%) over a given sample interval is calculated by a solute mass balance on the draw side:

(3) $$R\%=\left(1-\frac{V_{Out,D}C_{Out,D}-V_{In,D}C_{In,D}}{{A_{mem}J_{W}C}_{F,Avg}\;}\right)\times 100\%,$$

where *V_Out_*_,*D*_ and *V_In_*_,*D*_ denote the volumetric flow rate (L/h) of draw solution of the outlet and inlet, respectively, *C_Out_*_,*D*_ and *C_In_*_,*D*_ are the concentrations of the rejected feed element in the draw solution of the outlet and inlet, respectively, and *C_F_*_,*Avg*_ is the average concentration of the inlet and outlet of the feed element in the feed. Further details on the analysis of these metrics are presented in the Supplementary Materials.

### 2.5. Minimum Osmotic Differential

The minimum osmotic differential (Δ*π_min_*) indicates the minimum difference between the bulk feed and draw solution osmotic pressures. In the present work, Δ*π_min_* is used to parameterize the water flux. It is analogous to the temperature of approach in a heat exchanger, which defines the minimum temperature differential before heat transfer effectively ceases. As Δ*π_min_* is only measurable at the inlets and outlets, it occurs at one of two possible membrane positions in counter-current operation:

1. Feed-In (Feed) and Draw-Out (Diluted Draw Solution, DDS),
2. Feed-Out (Conc) and Draw-In (Concentrated Draw Solution, CDS).

Since it is ambiguous without measurement or calculation, which side of the membrane has reached this minimum, both sides are considered. For counter-current flow through the membrane module, the constraints for FO operation become:

(4) $$\Delta \pi_{min}\leq \pi_{DDS}-\pi_{Feed},$$

(5) $$\Delta \pi_{min}\leq \pi_{CDS}-\pi_{Conc},$$

where *π_i_* is the osmotic pressure of a stream (*i* = *Feed*, *Conc*, *DDS*, *CDS*) entering or leaving the FO unit operation.

The calculation of the minimum osmotic gradient is thus:

(6) $$\Delta \pi_{min}=\min\left(\pi_{DDS}-\pi_{Feed},\pi_{CDS}-\pi_{Conc}\right)$$

### 2.6. Measurement and Validation of Osmotic Pressures

The solutions studied in this work all had ionic strengths greater than 2 mol/L, so they were expected to have significant thermodynamic non-idealities associated with them. This means that the calculation of osmotic pressure requires either an activity coefficient model or a direct water activity measurement. To achieve less than 1 bar osmotic pressure accuracy, we chose to use a vapour pressure osmometer (LabMaster-aw Neo, Novasina, Lachen, Switzerland) with a resolution of 0.0001 water activity in the mole fraction scale (corresponding to ~0.1 bar osmotic pressure). To convert a water activity measurement to osmotic pressure, the following definition of osmotic pressure was used [31]:

(7) $$\pi =-\frac{RTln\left(a_{w}\right)}{{\hat{V}}_{w}},$$

where *π* is the osmotic pressure (bar), *R* is the gas constant (8.314 × 10^−5^ bar·m^3^/K·mol), *T* is the temperature (298 K), *a_w_* is the activity of water in the mole fraction scale, and ${\hat{V}}_{w}$ is the molar volume of pure water at 298 K (1.81 × 10^−5^ m^3^/mol).

**Figure 2:**
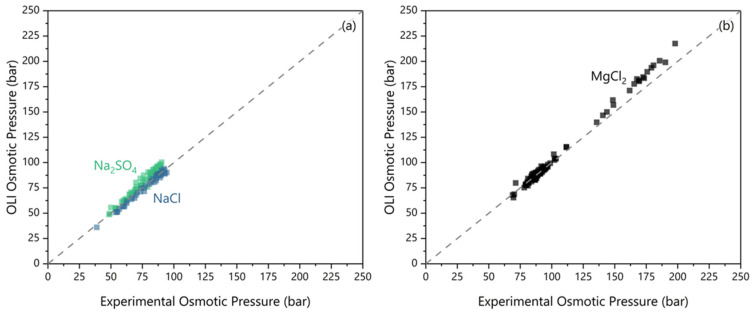
The measured osmotic pressure (via osmometer) versus OLI predicted osmotic pressure for (**a**) the feeds and (**b**) the draw.

The water activity measurements were compared to OLI’s Mixed Solvent Electrolyte (MSE) model V12.0 predictions (OLI Inc., Parsippany, NJ, USA). The validation results are presented in Figure 2 and reflect measurements from samples collected from the FO experiments. The concentrations used in the OLI simulation were from ICP measurements of electrolyte compositions of the same samples. The NaCl values agree with the measured values with a median deviation of −1.8 ± 2.0 bar osmotic pressure. With Na_2_SO_4_, the OLI predicted results deviated by 4.5 ± 3.5 bar. MgCl_2_ shows greater deviation depending on the osmotic pressure. At osmotic pressures less than 100 bar, a prediction error of −0.6 ± 2.0 bar was observed. At osmotic pressures higher than 100 bar, a clear linear deviation occurs for MgCl_2_. We consider this comparison as a demonstration of acceptable consistency between the OLI model predictions and the experimental measurements.

However, the variance of using ICP measurements with OLI to quantify osmotic pressures was not tolerable in this work due to the attempt to measure osmotic differentials near 0 bar. To avoid experimental error resulting in negative osmotic differentials, all osmotic differentials reported are from direct water activity measurements.

## 3. Results and Discussion

### 3.1. Na and S Rejection

FO experiments were carried out using the conditions of Table 2. These conditions were selected to represent the typical limit of RO water removal from a saline feed, and both feed and draw solutions were adjusted to pH 5.5 to match the natural pH of MgCl_2_ [17]. During the experiment, the recirculating draw solution became diluted, while the feed became more concentrated. This dilution of the draw solution and concentration of the feed caused the osmotic pressures of both solutions to approach each other, resulting in progressively lower water fluxes until reaching an endpoint near 0 L/m^2^h (LMH). According to Table 2, the experiment’s endpoint yielded NaCl concentrations of 9.7 ± 0.2 wt% or Na_2_SO_4_ concentrations of 21.2 ± 0.2 wt%, and MgCl_2_ DDS concentrations of 10.3 ± 0.1 wt% or 10.4 ± 0.1 wt%, respectively. Due to the acid concentration, the Na_2_SO_4_ and NaCl feeds decreased to 5.1 and 5.3, respectively. The final Na_2_SO_4_ concentration is close to its 21.5 wt% solubility limit at 25 °C, representing the first known instance of a commercial FO membrane saturating Na_2_SO_4_ [32].

**Figure 3:**
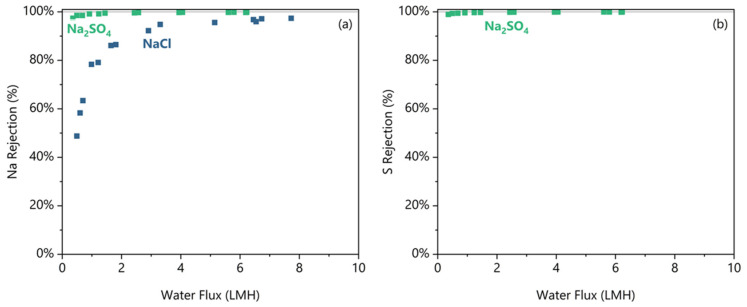
The (**a**) Na rejection and (**b**) S rejection plotted against the water flux for the pH 5.5 condition for NaCl and Na_2_SO_4_ feed solutes. Duplicates shown as separate data points.

By periodic sampling during the experiment, the FO performance at decreasing water fluxes was measured, and the rejection data are shown in Figure 3. Figure 3a shows that Na was better rejected from the Na_2_SO_4_ feed than the NaCl feed at any water flux; the greater rejection occurred even though the Na concentration was 2–3 times higher on average in the Na_2_SO_4_ feed. Critically, both feeds exhibited decreasing rejections below a minimum water flux. The decrease in the rejections is caused by both an increase in the feed solute concentration, which increases the salt flux, and the simultaneous decrease in the osmotic differential, which reduces the water flux. For detailed calculations determining this minimum water flux threshold, see the Supplementary Materials. Na_2_SO_4_ shows 99.7 ± 0.1% Na rejection at any water flux higher than 2.9 LMH (all errors at 95% confidence). Below the 2.9 LMH threshold, the Na rejection from Na_2_SO_4_ drops as low as 97% at 0.4 LMH. For NaCl, the rejection decreases from 95.8 ± 1.7% above its 3.3 LMH threshold to as low as 49% at 0.5 LMH. For the anions, Cl rejection was not measured reliably as explained below. From Figure 3b, the S rejection is shown to match the Na rejection trend in the Na_2_SO_4_ case, and S is 99.9 ± 0.1% rejected at water fluxes above 2.3 LMH.

**Figure 4:**
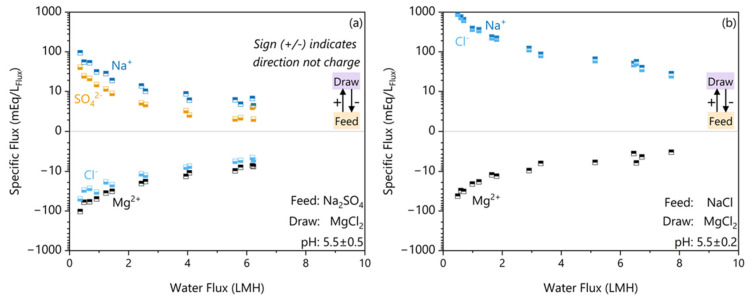
The specific fluxes per ion in absolute electron equivalents (mEq/L_FLux_) plotted against the water flux for feed solutes (**a**) Na_2_SO_4_ and (**b**) NaCl. The direction of transfer is indicated by the sign, with the feed to the draw solution being the positive direction.

The rejection results allude to the anion rejection being a limiting factor on the Na rejection due to the need to maintain electroneutrality. This membrane has a zeta potential of −12 mV with 1 mM KCl at pH 5.5 [21] indicating a negatively charged surface. While the ionic strengths used in these experiments will drastically reduce the absolute magnitude of the zeta potential, the membrane will still be negatively charged at high ionic strengths [33]. This negative surface charge repels the anion and, through electroneutrality, can limit the cation. Since a SO_4_^2−^ ion carries more charge and is larger than a Cl^−^ ion, we postulate that it is expected that sodium flux will be smaller in the Na_2_SO_4_ feed than in the Cl^−^ due to electroneutrality restrictions. To confirm, we had to also measure the anion fluxes, which is impossible in the NaCl feed due to the masking effect of the draw salt and very difficult to measure accurately in the Na_2_SO_4_ feed due to the high ionic strengths. Instead, the Cl^−^ flux was estimated by a charge balance. To gain a clearer look at the individual ion fluxes, Figure 4 plots the specific ion fluxes in terms of their absolute charge equivalence that resulted in the rejections shown in Figure 3.

The Na_2_SO_4_ ion flux results in Figure 4a show that the flux of Na^+^ across the membrane is 56% greater than the charge equivalent amount of SO_4_^2−^ flux. To maintain electroneutrality, Na^+^ permeation requires either forward flux of SO_4_^2−^ or the reverse transfer of Mg^2+^. The hydrated radius of SO_4_^2−^ (521 ± 7 pm) and Mg^2+^ (626 ± 4 pm) are both significantly larger than Na^+^ (375 ± 6 pm) [34]. In a porous FO membrane, a larger hydrated size causes greater resistance to salt transfer due to the need to release more water of hydration before permeating [35,36,37]. Since both options are limited by hydrated size exclusion, the Na^+^ flux is electroneutrality-limited by the bidirectional ion permeation. These results of electroneutrality-limited bidirectional permeation of ions in FO agree with the theory and modelling of Hancock et al. [38] in flat sheet systems. The electroneutrality-limited Na^+^ flux also agrees with the experimental findings of Arena et al. [39], which shows an increase in bidirectional cation transfer when the feed solute is present versus not.

In contrast, the NaCl case shown in Figure 4b shows that Na^+^ and Cl^−^ fluxes were in the same direction. The hydrated radius of Cl^−^ (458 ± 7 pm) is significantly smaller than the hydrated radius of SO_4_^2−^ (521 ± 7 pm), which explains why Cl^−^ passed more easily through the porous FO membrane in the NaCl case [34,36,37]. The absence of a limiting counter-ion explains the lower rejection despite the 2–3 times lower Na^+^ concentration differential.

### 3.2. Reverse Draw Solute Flux

**Figure 5:**
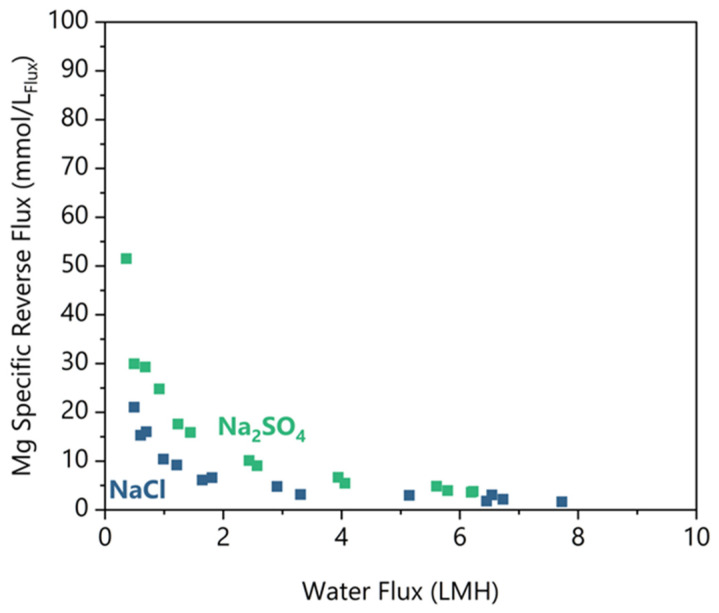
Mg-specific reverse salt flux rejection plotted against the water flux for the pH 5.5 condition for NaCl and Na_2_SO_4_ feed solutes. Duplicates shown as separate data points.

Reverse Mg diffusion is a problem because it represents a reagent loss and contamination of the concentrated feed, which could complicate any downstream processes. Figure 5 shows the specific reverse Mg flux for Na_2_SO_4_ and NaCl feed salts. Similarly to the Na Rejection, there is a minimum water flux threshold below which Mg flux increases exponentially. For Na_2_SO_4_ above 2.8 LMH, the specific flux was 5.3 ± 3.1 mmol_Mg_/L_Flux_, and for NaCl above 3.2 LMH, the specific flux was 2.6 ± 1.0 mmol_Mg_/L_Flux_ (at 95% confidence). Below their threshold water fluxes, both feeds demonstrated an increase in reverse Mg flux, but Na_2_SO_4_ demonstrated a 2 times greater increase than NaCl at equivalent water fluxes.

There are limited studies available to compare with those that used similar solutes and concentrations. In a Li brine concentration study by Pham et al. [8] a simulated lake brine draw solution included 0.49–0.77 mol_Mg_/L, and their work concentrated LiCl from 0.43 to 1.8 mol_Li_/L. Importantly, performance testing with a flat sheet aquaporin membrane resulted in a 5 mmol_Mg_/L_Flux_ reverse flux with declining water fluxes from 5 to 0 LMH, similar to our results. Sanahuja–Embuena et al. [21] tested an HFFO2 module (identical to this study) in single-pass mode with a DI water feed and MgCl_2_ draw, which resulted in 0.3–0.5 mmol_Mg_/L_Flux_. Their water fluxes were significantly higher at 12–22 LMH. The higher water fluxes and lack of a feed salt are likely why their reverse Mg flux was much less. These works underscore the importance of both water flux and the presence of feed solutes on the reverse solute flux of Mg.

We can also observe in Figure 5 that the higher diffusion of Mg occurs when the water flux is less than 3.2 LMH. In addition, Na_2_SO_4_ results in two times higher reverse Mg flux compared to NaCl under equivalent water fluxes. We postulate that the higher water fluxes provide a convective counter to the Mg diffusion. Under the low water flux conditions (<3.2 LMH), the Mg draw concentration was 18–19% lower on average than in the high-water flux region. Since the reverse diffusion increased with a lower concentration differential, Mg concentration could not drive the increase in reverse salt flux. Thus, the remaining difference is the water flux. The water flux occurs in the opposite direction from the Mg flux, and the water flux must be reduced to the point where Mg can sufficiently overcome the convection to diffuse into the feed. As we have shown, it is necessary to operate the FO membrane at greater than 3.2 LMH to minimize Mg reverse draw flux.

To explain the higher reverse Mg flux with a Na_2_SO_4_ feed, we postulate that electroneutrality-limited bidirectional ion transfer enhances the reverse salt flux. Figure 4 shows that the net direction of Cl^−^ flux is in the reverse direction or parallel to the Mg flux. In addition, there is a 2–3 times higher Na concentration in the feed, unable to transfer across the membrane due to the need to charge balance SO_4_^2−^. There are three main pathways for Mg^2+^ reverse solute transport:

1. Mg^2+^ exchanges with 2 Na^+^ ions from the feed,
2. Mg^2+^ diffuses with one reverse Cl^−^ ion and exchanges with 1 Na^+^ ion,
3. Mg^2+^ diffuses with two reverse Cl^−^ ions.

Pathway 3 is the same regardless of feed solute, so it is not the cause of the difference. In the NaCl feed case, pathway 1 is more challenging, since there is a lower Na concentration. Additionally, pathway 2 is more likely in the Na_2_SO_4_ case due to the net direction of the Cl^−^ flux. Thus, we may conclude that the increase in Mg reverse flux in the Na_2_SO_4_ case is the combination of a higher feed solute concentration and the reverse direction of the draw anion flux.

### 3.3. Water Flux Versus Minimum Osmotic Differential

**Figure 6:**
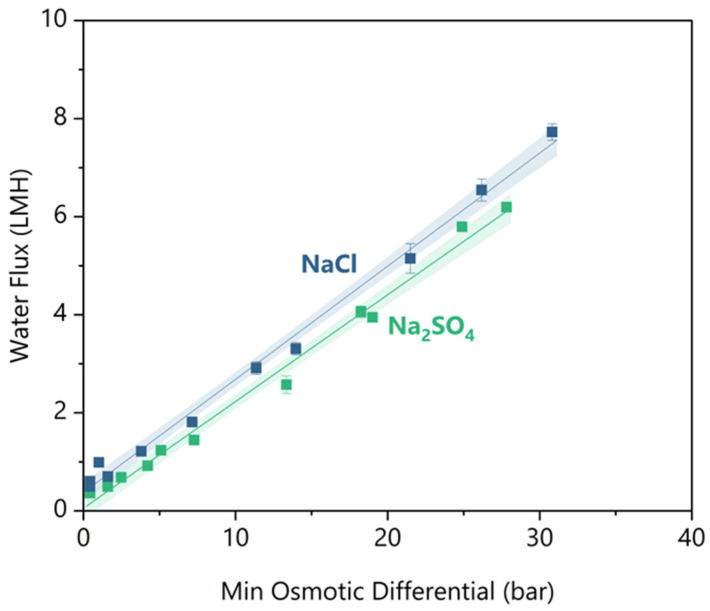
The water flux versus minimum osmotic gradient for feed solutes of NaCl and Na_2_SO_4_. Duplicates are shown as individual datapoints, and the error bars indicate uncertainty in the water flux measurement between the 2 balances. Both solutes have linear regression fitted, and the shaded region indicates a 95% confidence interval.

So far, we have shown that there is a sharp decrease in rejections and an increase in reverse draw solute flux below a minimum water flux. This trend demonstrates the inability to simply look up separation efficacy values for a given membrane, as process conditions can significantly influence the separation efficacy. Instead, we need a parameter to specify operating conditions that ensure sufficient water flux while maintaining separation efficacy. For this parameter, we defined Δ*π_min_* as follows: a performance rule of thumb, analogous to “temperature of approach” in a heat exchanger (see definition in Section 2.5). The linearity of Δ*π_min_* with respect to water flux can be observed in Figure 6. This linearity allows for estimating water flux performance based on properties that can be calculated within a mass balance, eliminating the need for kinetics modelling.

Figure 6 shows the water flux results for synthetic feeds of NaCl and Na_2_SO_4_ measured at decreasing minimum osmotic differential. At greater than 5 bar minimum osmotic differential, NaCl had ~1 LMH greater water flux than Na_2_SO_4_. As the feed concentrates from 50 to 90 bar osmotic pressure, NaCl is 2 to 2.5 times more diffusive than Na_2_SO_4_ (per OLI MSE V12) [40]. The higher diffusivity means that NaCl has half the mass transfer resistance of Na_2_SO_4_, so NaCl experiences lower concentration polarization on the lumen side of the fibre [41]. The lower concentration polarization increased the effective osmotic differential at the membrane interface and caused the water flux to be higher at equivalent bulk osmotic differentials.

For practical FO design, it would be convenient to have a rule of thumb value for Δ*π_min_* that maintains effective water flux kinetics, high solute rejections, and minimal reverse solute flux. There is little guidance available to suggest what is sufficient water flux kinetics for FO, but some pilot studies have claimed successful operations with ~3 LMH [42,43,44]. As discussed in the previous sections, there are minimum threshold water fluxes before an exponential loss of rejection or an increase in reverse salt flux. However, the greater the minimum threshold value, the greater the osmotic driving force. The greater the osmotic driving force, the higher the concentration of the draw solution that will be required, which will increase material costs and the energy of the draw regeneration. Therefore, using the minimum water flux threshold is beneficial. To ensure separation is maintained, the largest of the threshold water fluxes across performance metrics should be considered the minimum required water flux. For Na_2_SO_4_, 2.8 LMH is required to prevent Mg reverse flux increase, but for NaCl, 3.3 LMH is required to prevent a loss of Na Rejection. Using these minimum water fluxes and the linear trend in Figure 6, the required minimum osmotic differentials are 12.7 ± 0.7 bar and 12.6 ± 0.6 bar for NaCl and Na_2_SO_4_, respectively. Effectively, there is no difference in the minimum osmotic differential requirement for the two salts.

### 3.4. Limited pH Effect on FO Performance

Our objective is to demonstrate that current commercial FO membranes can dewater hypersaline NaCl and Na_2_SO_4_ brines. As discussed in the introduction, when these brines originate from hydrometallurgical streams, they are often acidic, so the final step is to demonstrate the robustness of separation efficacy against low pH.

In this section, the same experimental procedure was used except that the initial pH of the feed and draw solution was varied to 3, 5.5, and 7. These were chosen to remain within the operating specifications of the HFFO2 module. Due to the nature of the experiments, no adjustment could be made throughout the trial, so as the feed was concentrated, its pH decreased, and as the draw was diluted, its pH increased. The pH results are summarized in Table 3 alongside the expected pH change predicted by OLI Studio (MSE V12.0). The OLI Studio simulations quantify any pH changes solely due to changes in concentration and ion activity. Deviations from the predicted pH values indicate proton transfer, and the membrane was not anticipated to be selective for H_3_O^+^ due to its small size (275 ± 1 pm) [34].

The rejection of the anion and the need for electroneutrality resulted in concentrating dilute sulphuric acid in the feed. Since the draw in the Na_2_SO_4_ case was diluted without proton transfer, its pH rose. Conversely, in the NaCl case, the feed pH does not decrease as much as predicted. There is significantly more anion transfer with NaCl feeds, so there is no electroneutrality limitation on proton transfer. In this case, the draw dilutes with greater proton transfer, so the pH is lower than predicted. While the pH variation was a challenge for these experiments, it identified a potential method for upgrading dilute sulphuric acid streams.

**Figure 7:**
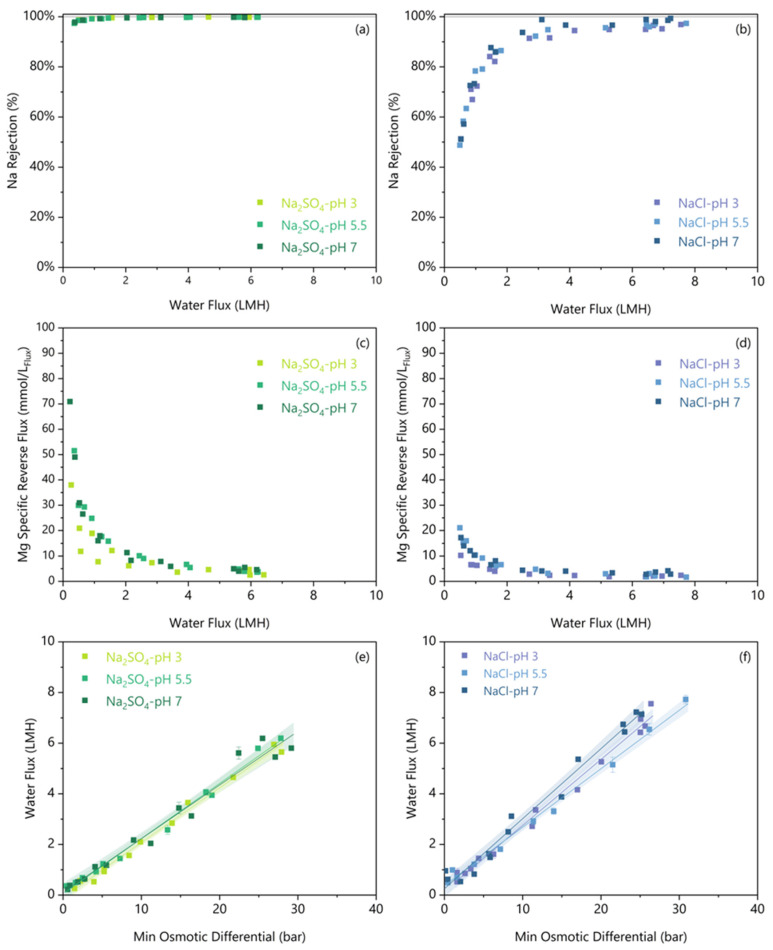
The Na rejection (**a**,**b**) and Mg specific reverse salt flux (**c**,**d**) are plotted against the water flux for varied initial pH and feed solutes. The water flux versus minimum osmotic gradient for varied initial pH and feed solutes (**e**,**f**). A linear regression is fitted on a per-solute/pH condition, and the shaded region indicates a 95% confidence interval.

No significant difference was observed between any of the pHs concerning the Na Rejection (Figure 7a,b) or S Rejection (Supplementary Materials) for either salt. The Na rejections above the threshold water flux for all tests were compared in Table 4, and there were no significant differences in Na rejections for a given feed solute at varying pH. The neutral pH 7 condition has a 0.3–0.5 LMH lower threshold water flux before rejection decreases for both feed salts. A similar work on flat sheet FO membranes found no effect of pH from 1 to 7 on Li rejection [8]. Their work also used a MgCl_2_ draw and a concentrated Li brine above 0.43 mol_Li_/L. From our work, the consistent rejections at varying pH demonstrate rejection robustness to pH as low as 3.

Reverse Mg flux was higher under pH 7 than pH 3 only for NaCl, as shown in Figure 7c,d, and Table 4. Between pH 3 and pH 7, Na_2_SO_4_ showed no significant difference in reverse Mg flux, but NaCl showed a marginal 1.7 ± 1.0 mmol_Mg_/L_Flux_ increase. Alongside this higher reverse salt flux at pH 7, the minimum water flux threshold increases by up to 1 LMH for NaCl. However, for both feed solutes, the pH 5.5 and pH 3 reverse Mg fluxes are not significantly different, and above the threshold water flux, all reverse Mg fluxes remain low, below 10 mmol_Mg_/L_Flux_. Thus, we observed a minimal effect of pH on reverse salt flux.

The more acidic solution can protonate the carboxyl surface groups on the membrane, which would hinder cation transfer [39]. Arena et al. [39] showed a ~4 times increase in reverse draw solute flux when the pH increased from 2 to 6; however, they used a KCl draw solution instead of MgCl_2_. The solutions in this study were at significantly higher ionic strengths (above 2 mol/L), which reduced the thickness of the diffuse double layer, limiting its effect. Overall, the slightly lower reverse Mg flux at acidic pH demonstrates reverse salt flux robustness to pH as low as 3.

The relationship between water flux and the minimum osmotic gradient for both Na_2_SO_4_ and NaCl was unaffected by the feed pH, as shown in Figure 7e,f. The literature presents conflicting results regarding the effect of pH on water flux through polyamide membranes. Similarly to our findings, Arena et al. [39] found no significant changes in water flux with respect to pH when using a polyamide membrane. Conversely, Pramanik et al. [7] examined the effect of pH on water flux using a different polyamide membrane (Porifera, San Leandro, CA, USA) and discovered that lower pH conditions reduced water flux. Critically, since their membrane was a flat sheet, they were able to perform contact angle measurements to show that the active layer contact angle increased from 49.5 ± 1.4° at pH 7 to 61.2 ± 1.4° at pH 3 [7]. This higher contact angle measured under acidic pH demonstrated that the membrane hydrophobicity increases, which should result in a lower water flux. However, our study did not observe any significant change in water flux under acidic conditions over short-duration experiments. Nevertheless, it is possible that long-duration tests may detect a change.

Taking into consideration all the small pH effects, Table 4 shows that a ~1 bar higher minimum osmotic differential is required under acidic conditions to limit Na Rejection decreases. At pH 3, the minimum osmotic differential is 14.0 ± 0.8 bar for Na_2_SO_4_ and 13.5 ± 0.8 bar for NaCl. There remains no significant difference in osmotic requirements between salts, even at different pH levels. Importantly, this increase is trivial compared to the typical error of quantifying osmotic pressures of 2–5 bar (see Section 2.6). Thus, as a conservative rule of thumb, a Δ*π_min_* of 15 bar is a reasonable starting assumption for an FO operation to maintain sufficient water flux without suffering decreases in membrane rejection or increases in reverse salt flux. This engineering parameter allows the calculation of the steady state required draw solution concentration and flow for scenarios where FO performance data is unavailable.

## 4. Conclusions

This work demonstrated the efficacy of commercial FO dewatering hypersaline feeds of NaCl and Na_2_SO_4_ using a MgCl_2_ draw over equivalent osmotic conditions. We found that the rejections of feed contaminants strongly depend on the operating conditions, especially the water flux. A 95.8% NaCl rejection requires at least 3.3 LMH of water flux, below which it decreases to as low as 49%. Na_2_SO_4_ is rejected 99.7% at greater than 2.1 LMH and only suffers a modest reduction in Na rejection to 97%. The differences in solute transport were attributed to the hydrated size of SO_4_^2−^ and Mg^2+^, which, due to electroneutrality, limited the permeation of Na^+^ in the Na_2_SO_4_ case. Similarly, minimum water flux thresholds were identified for reverse solute flux of Mg for both salts. Additionally, Na_2_SO_4_ showed twice the reverse Mg flux as NaCl. We identified that both the direction of anion transfer and the concentration of the Na^+^ ions were likely responsible for the difference in reverse flux.

We defined a minimum osmotic differential as a way to parameterize FO dewatering performance without relying on advanced kinetic models to simplify process modelling efforts. For a given membrane, this parameter value can be determined experimentally by: (1) acquiring rejection and reverse salt flux data as a function of water flux; (2) determining the minimum water flux threshold below which either rejections or reverse salt fluxes change exponentially; (3) using the linear relationship between water flux and minimum osmotic differential to determine the appropriate minimum osmotic differential. For example, at pH 3, the HFFO2 module requires 2.9 LMH and 3.7 LMH for Na_2_SO_4_ and NaCl, respectively. This meant NaCl required a minimum osmotic differential of 13.5 ± 0.8 bar, while Na_2_SO_4_ required 14.0 ± 0.8 bar. Since this parameter difference between solutes is insignificant, we generalized that a conservative 15 bar minimum osmotic differential is useful for cases where experimental data is not yet available.

The FO membrane investigated is robust to changes in pH from 3 to 7. Over this range, there are no impactful changes in separation efficacy or in the relationship between water flux versus osmotic pressure differential. Ultimately, this work showed that existing commercial FO membranes can dewater hypersaline Na_2_SO_4_ brines to 98.6% saturation effectively.

## Figures and Tables

**Table 1:** A summary of FO membranes commercially available in 2025.

Manufacturer, City, Country	Membrane Material	Membrane Geometry	Module Area (m^2^)	pH Tolerance	Reference
Aquaporin, Kongens Lyngby, Denmark	Polyamide	Hollow Fibre	2.3, 13.8	3–10	[26]
Porifera, San Leandro, CA, USA	Polyamide	Flat Sheet	N/A *	2–11	[14,27]
Toyobo, Osaka, Japan	Cellulose Triacetate	Hollow Fibre	31.5	3–8	[28]
Fluid Technology Solutions, Albany, NY, USA	Cellulose Triacetate	Flat Sheet/ Spiral Wound	N/A */ 13.0, 21.5	3–7	[29]

* Flat Sheets do not have a set module area.

**Table 2:** Experimental conditions. Each were similar for the pH = 3, pH = 5.5, and pH = 7 tests.

Feed						Draw					
Solute	Initial Conc.	Initial Osmotic Pressure	Inlet Flow Rate	Final Conc.	Final Osmotic Pressure	Solute	Initial Conc.	Initial Osmotic Pressure	Inlet Flow Rate	Final Conc.	Final Osmotic Pressure
	(wt%)	(bar)	(L/min)	(wt%)	(bar)		(wt%)	(bar)	(L/min)	(wt%)	(bar)
Na_2_SO_4_	12.9	51	0.8 ± 0.1	21.2 ± 0.2	91 ± 1	MgCl_2_	13.8	175	0.5 ± 0.1	10.3 ± 0.1	104 ± 1
NaCl	5.9	50	0.8 ± 0.1	9.7 ± 0.2	90 ± 3	MgCl_2_	14.6	195	0.5 ± 0.1	10.4 ± 0.1	105 ± 1

**Table 3:** The initial and final pH per solute and the predicted final pH via OLI Studio (OLI MSE V12.0).

Feed				Draw			
Solute	Measured Initial pH	Measured Final pH	Predicted Final pH	Solute	Measured Initial pH	Measured Final pH	Predicted Final pH
Na_2_SO_4_	3.0 ± 0.1	2.9 ± 0.1	2.8	MgCl_2_	3.1 ± 0.1	4.3 ± 0.1	3.3
	5.6 ± 0.1	5.1 ± 0.1	5.3		5.5 ± 0.1	6.1 ± 0.1	5.8
	7.0 ± 0.1	6.1 ± 0.1	6.9		7.0 ± 0.1	7.2 ± 0.1	7.2
NaCl	3.0 ± 0.1	2.8 ± 0.1	2.5	MgCl_2_	3.0 ± 0.1	3.1 ± 0.1	3.4
	5.6 ± 0.1	5.3 ± 0.1	5.1		5.4 ± 0.1	5.6 ± 0.1	5.8
	7.1 ± 0.1	6.8 ± 0.1	7.0		7.0 ± 0.1	7.2 ± 0.1	7.3

**Table 4:** Summary of FO metrics, minimum water flux requirements, and the resulting minimum osmotic differentials.

Solute	Initial pH	Na Rejection (%)	Min. Water Flux * (LMH)	Specific Mg Flux (mmol_Mg_/L_Flux_)	Min. Water Flux ** (LMH)	Δπ_min_ (bar)
Na_2_SO_4_	3.0	99.7 ± 0.1	2.94	5.2 ± 3.1	1.54	14.0 ± 0.8
	5.5	99.7 ± 0.1	2.14	5.3 ± 3.1	2.82	12.7 ± 0.7
	7.0	99.7 ± 0.1	2.38	8.1 ± 2.4	1.74	10.7 ± 1.1
NaCl	3.0	95.0 ± 1.7	3.68	2.1 ± 0.3	2.25	13.5 ± 0.6
	5.5	95.8 ± 1.7	3.28	2.6 ± 1.0	3.25	12.6 ± 0.6
	7.0	97.2 ± 1.6	3.31	3.8 ± 0.7	3.78	11.6 ± 0.8

The error shown is a 95% confidence interval. * Min. Water Flux corresponding to Na Rejection in the column to the left. ** Min. Water Flux corresponding to Specific Mg Flux in the column to the left.

## Data Availability

The data presented in this study are available on request from the corresponding author due to standard practice for industrial partner privacy and intellectual property protection.

## Supplementary Materials

The following supporting information can be downloaded at: https://www.mdpi.com/article/10.3390/membranes16010014/s1, Table S1. Properties of seawater and hypersaline brines at 25 °C. Figure S1. Examples of determining the minimum water flux for Na Rejection and Specific Mg Flux. Figure S2. The S rejection plotted against the water flux for varied initial pH with Na_2_SO_4_ as the feed. Figure S3. Sample time series data.

## References

[1] Binnemans K., Jones P.T. (2023). The Twelve Principles of Circular Hydrometallurgy. J. Sustain. Metall..

[2] Devaere N., Papangelakis V. (2023). Forward Osmosis for Metal Processing Effluents under Similar Osmotic Pressure Gradients. Membranes.

[3] Shah K.M., Billinge I.H., Chen X., Fan H., Huang Y., Winton R.K., Yip N.Y. (2022). Drivers, challenges, and emerging technologies for desalination of high-salinity brines: A critical review. Desalination.

[4] Choi J.-W., Shim H.-W., Kim H.-I., Kim S., Tho Tran D., Bae M. (2025). Toward closed-loop hydrometallurgy: A critical review of wastewater reuse strategies for end-of-life LiFePO 4 battery recycling. Green Chem..

[5] Matebese F., Mosai A.K., Tutu H., Tshentu Z.R. (2024). Mining wastewater treatment technologies and resource recovery techniques: A review. Heliyon.

[6] Kolliopoulos G., Xu C., Martin J.T., Devaere N., Papangelakis V.G. (2022). Hybrid forward osmosis—Freeze concentration: A promising future in the desalination of effluents in cold regions. J. Water Process Eng..

[7] Pramanik B.K., Shu L., Jegatheesan J., Shah K., Haque N., Bhuiyan M.A. (2019). Rejection of rare earth elements from a simulated acid mine drainage using forward osmosis: The role of membrane orientation, solution pH, and temperature variation. Process Saf. Environ. Prot..

[8] Pham M.T., Nishihama S., Yoshizuka K. (2020). Concentration of lithium by forward osmosis. Hydrometallurgy.

[9] Baena-Moreno F.M., Rodríguez-Galán M., Arroyo-Torralvo F., Vilches L.F. (2020). Low-Energy Method for Water-Mineral Recovery from Acid Mine Drainage Based on Membrane Technology: Evaluation of Inorganic Salts as Draw Solutions. Environ. Sci. Technol..

[10] Rahbari-Sisakht M., Ismail A.F. (2025). A comprehensive review of pressure and osmosis driven membrane processes: Processes, characteristics and materials. Desalination.

[11] Blandin G., Ferrari F., Lesage G., Le-Clech P., Héran M., Martinez-Lladó X. (2020). Forward Osmosis as Concentration Process: Review of Opportunities and Challenges. Membranes.

[12] Mahto A., Aruchamy K., Meena R., Kamali M., Nataraj S.K., Aminabhavi T.M. (2021). Forward osmosis for industrial effluents treatment—Sustainability considerations. Sep. Purif. Technol..

[13] Shaffer D.L., Werber J.R., Jaramillo H., Lin S., Elimelech M. (2015). Forward osmosis: Where are we now?. Desalination.

[14] Abounahia N., Ibrar I., Kazwini T., Altaee A., Samal A.K., Zaidi S.J., Hawari A.H. (2023). Desalination by the forward osmosis: Advancement and challenges. Sci. Total Environ..

[15] Ferella F., Suichies A., Abdelkader B.A., Dabhi N.K., Werber J., de Lannoy C.-F. (2025). Ocean Alkalinity Enhancement Using Bipolar Membrane Electrodialysis: Technical Analysis and Cost Breakdown of a Full-Scale Plant. Ind. Eng. Chem. Res..

[16] Fang Z., Zhu P., Zhang X., Feng Y., Wang H. (2025). Self-looped electrochemical recycling of lithium-ion battery cathode materials to manufacturing feedstocks. Nat. Chem. Eng..

[17] Baker R.W. (2004). Membrane Technology and Applications.

[18] McCutcheon J.R., Elimelech M. (2006). Influence of concentrative and dilutive internal concentration polarization on flux behavior in forward osmosis. J. Membr. Sci..

[19] Yalamanchili R., Cegarra P.O., Galizia A., Rodriguez-Roda I., Blandin G. (2025). Single-pass forward osmosis for efficient feed concentration: Optimizing multiple modules arrangement and flow distribution. Desalination.

[20] Sbardella L., Blandin G., Fàbregas A., Carlos Real Real J., Serra Clusellas A., Ferrari F., Bosch C., Martinez-Lladó X. (2022). Optimization of pilot scale forward osmosis process integrated with electrodialysis to concentrate landfill leachate. Chem. Eng. J..

[21] Sanahuja-Embuena V., Khensir G., Yusuf M., Andersen M.F., Nguyen X.T., Trzaskus K., Pinelo M., Helix-Nielsen C. (2019). Role of Operating Conditions in a Pilot Scale Investigation of Hollow Fiber Forward Osmosis Membrane Modules. Membranes.

[22] Awad A.M., Jalab R., Minier-Matar J., Adham S., Nasser M.S., Judd S.J. (2019). The status of forward osmosis technology implementation. Desalination.

[23] Jun B.M., Kim S.H., Lim H.Y., Kwak S.K., Kwon Y.N. (2022). Acid stability of polyamide membranes. Polymer.

[24] Porter C.J., Werber J.R., Zhong M., Wilson C.J., Elimelech M. (2020). Pathways and Challenges for Biomimetic Desalination Membranes with Sub-Nanometer Channels. ACS Nano.

[25] Jie Lim Y., Goh K., Wang R. (2022). The coming of age of water channels for separation membranes: From biological to biomimetic to synthetic. Chem. Soc. Rev..

[26] Aquaporin A/S (2025). Aquaporin Inside HFFO2 Datasheet. https://aquaporin.com/products/aquaporin-inside-hffo2/.

[27] Suwaileh W., Pathak N., Shon H., Hilal N. (2020). Forward osmosis membranes and processes: A comprehensive review of research trends and future outlook. Desalination.

[28] Toyobo MC Corporation (2023). TOYOBO MC Membrane Module for Forward Osmosis. https://www.toyobo-mc.jp/wordpress/wp-content/uploads/2023/10/Brochure_TMC_FO.pdf.

[29] Fluid Technology Solutions Inc. (2018). OsmoF2OTM FO Industrial Membrane. https://ftsh2o.com/wp-content/uploads/2018/07/FTS_FO_Industrial-Membrane_FINAL-FTSCTA-02.pdf.

[30] Towler G., Sinnott R., Towler G., Sinnott R. (2022). Chapter 3—Utilities and energy-efficient design. Chemical Engineering Design.

[31] Borgnakke C., Sonntag R.E. (2012). Fundamentals of Thermodynamics.

[32] Lide D.R., Rumble J.R., Bruno T.J. (2019). CRC Handbook of Chemistry and Physics: A Ready-Reference Book of Chemical and Physical Data.

[33] Coday B.D., Luxbacher T., Childress A.E., Almaraz N., Xu P., Cath T.Y. (2015). Indirect determination of zeta potential at high ionic strength: Specific application to semipermeable polymeric membranes. J. Membr. Sci..

[34] Marcus Y. (1988). Ionic radii in aqueous solutions. Chem. Rev..

[35] Tansel B. (2012). Significance of thermodynamic and physical characteristics on permeation of ions during membrane separation: Hydrated radius, hydration free energy and viscous effects. Sep. Purif. Technol..

[36] Song L., Heiranian M., Elimelech M. (2021). True driving force and characteristics of water transport in osmotic membranes. Desalination.

[37] Wang L., He J., Heiranian M., Fan H., Song L., Li Y., Elimelech M. (2023). Water transport in reverse osmosis membranes is governed by pore flow, not a solution-diffusion mechanism. Sci. Adv..

[38] Hancock N.T., Phillip W.A., Elimelech M., Cath T.Y. (2011). Bidirectional Permeation of Electrolytes in Osmotically Driven Membrane Processes. Environ. Sci. Technol..

[39] Arena J.T., Chwatko M., Robillard H.A., McCutcheon J.R. (2015). pH Sensitivity of Ion Exchange through a Thin Film Composite Membrane in Forward Osmosis. Environ. Sci. Technol. Lett..

[40] Anderko A., Wang P., Rafal M. (2002). Electrolyte solutions: From thermodynamic and transport property models to the simulation of industrial processes. Fluid Phase Equilibria.

[41] Khan M.A.W., Zubair M.M., Saleem H., AlHawari A., Zaidi S.J. (2024). Modeling of osmotically-driven membrane processes: An overview. Desalination.

[42] Werber J.R., Deshmukh A., Elimelech M. (2016). The Critical Need for Increased Selectivity, Not Increased Water Permeability, for Desalination Membranes. Environ. Sci. Technol. Lett..

[43] McGinnis R.L., Hancock N.T., Nowosielski-Slepowron M.S., McGurgan G.D. (2013). Pilot demonstration of the NH_3_/CO_2_ forward osmosis desalination process on high salinity brines. Desalination.

[44] Agrawal V., Kharade S., Achari M., Pawar H., Sarode D. (2024). Forward Osmosis: An Energy-Efficient Approach for the Treatment and Recovery of Resources from the Dairy Industry Effluent: Forward Osmosis Treatment of Dairy Industry Waste Stream Whey. J. Sci. Ind. Res. JSIR.

