# Supplementary material for: Dewatering Hypersaline Na2SO4 and NaCl via Commercial Forward Osmosis Module
Source: Membranes (Basel). 2025 Dec 31;16(1):14. doi: 10.3390/membranes16010014 (PMC12844510; doi:10.3390/membranes16010014)
Supplement: Supplementary file 1 [file membranes-16-00014-s001.zip › membranes-3972814-supplementary.pdf]

Supplementary Material: Dewatering Hypersaline Na_2SO_4 and NaCl via Commercial Forward Osmosis Module

Noel Devaere and Vladimiros G. Papangelakis *

Department of Chemical Engineering and Applied Chemistry, University of Toronto,
200 College St., Toronto, ON, Canada M2S 3E5
*Corresponding author

Physical Properties Comparison of Hypersaline Feed Solutions with Seawater

Table S1. Properties of seawater and hypersaline brines at 25 °C (Data from OLI Studio V12.0).

Property	Unit	NaCl 3.5 wt% (Seawater)	NaCl 5.9 wt% (RO Brine)	NaCl 9.7 wt% (=[1]Saturated Na_2SO_4)	Na_2SO_4 12.9 wt% (=[1]RO Brine)	Na_2SO_4 21.5 wt% (Saturated Na_2SO_4)
Osmotic Pressure	bar	28	50	90	51	92
Density	kg/m^3	1022	1039	1067	1113	1196
Absolute Viscosity	Pa•s	9.43×10^{-4}	9.85×10^{-4}	1.07×10^{-3}	1.40×10^{-3}	2.12×10^{-3}
[2]Average Diffusivity	m^2/s	1.47×10^{-9}	1.43×10^{-9}	1.35×10^{-9}	7.33×10^{-10}	5.59×10^{-10}
Foulant Solubilities						
$CaSO_4$	wt%	0.51%	0.60%	0.66%	0.19%	0.18%
[3]$CaCO_3$	wt%	0.085%	0.088%	0.083%	0.38%[4]	0.50%[4]

[1] '=' implies similar osmotic pressure as sample name, but using a different solute

[2] Average diffusivity is a geometric mean of the ion diffusivities, where each ion is raised to its relative abundance. $D_{NaCl}=(D_{Na}D_{Cl})^{1/2}$, $D_{Na2SO4}=(D_{Na}^2D_{SO4})^{1/3}$

[3] $CaCO_3$ (calcite) solubility calculated at pH 7 controlled with acid addition (HCl for NaCl, H_2SO_4 for Na_2SO_4).

[4] $CaSO_4{\cdot}2H_2O$ forms alongside $CaCO_3$.

Determination of Minimum Water Flux

Na Rejection (R_{Na}) and Specific Mg Flux (J_S/J_W) were observed to experience a plateau of near-constant performance, followed by an exponential change as the water flux (J_W) approached 0. To determine the point where the exponential change began, the following procedure was used to avoid inconsistency in graphical interpretation.

First, an asymptotic regression model was fit of the form Eq. (S1):

$$y = a - bc^{J_W}, \qquad (S1)$$

where a, b, and c are fitting constants and y is either performance metric (R_{Na} or J_S/J_W). This function was used as it is the simplest regression approach that contains an exponential and an asymptote term. Both terms are required for rejections, which have a maximum of 100%, and reverse slat flux, which has a minimum of 0 mmol/L. The asymptotic regression fits both reverse salt flux and the Na rejections well (R^2>0.93). While this is an empirical interpretation method, the '$a \pm a_{error}$' fitting constant is the expected membrane performance when operating above the minimum water flux (the y-value of the asymptote as J_W increases, provided $c < 1$). The 'b' value determines the magnitude of change in the performance metric at water fluxes below the minimum water flux. The 'c' value ultimately determines the rate of change in the performance metric as the water flux decreases.

Second, to find the threshold minimum water flux before the performance changes exponentially, y in Eq. (S1) is set to $a \pm a_{error}$. The a_{error} is the standard error of the fitting parameter from the regression. The minimum water flux ($J_{W,min}$) threshold where exponential change begins is now calculated from S2:

$$J_{W,min} = \frac{\ln\left(\left|\frac{a_{error}}{b}\right|\right)}{\ln(c)}. \qquad (S2)$$

Eq. (S2) contains an absolute value because the sign of a_{error}/b must be positive. Only one of the possible y-values ($a \pm a_{error}$) yields a real solution.

The results of this approach for the pH 5.5 conditions are shown in **Figure S1** below. **Table 4** (see main text) summarizes the asymptotic separation performance and minimum water flux to avoid the exponential change of that metric. Since there are two performance metrics to consider, the larger minimum water flux is used to find the minimum osmotic gradient from its linear trendline with water flux.

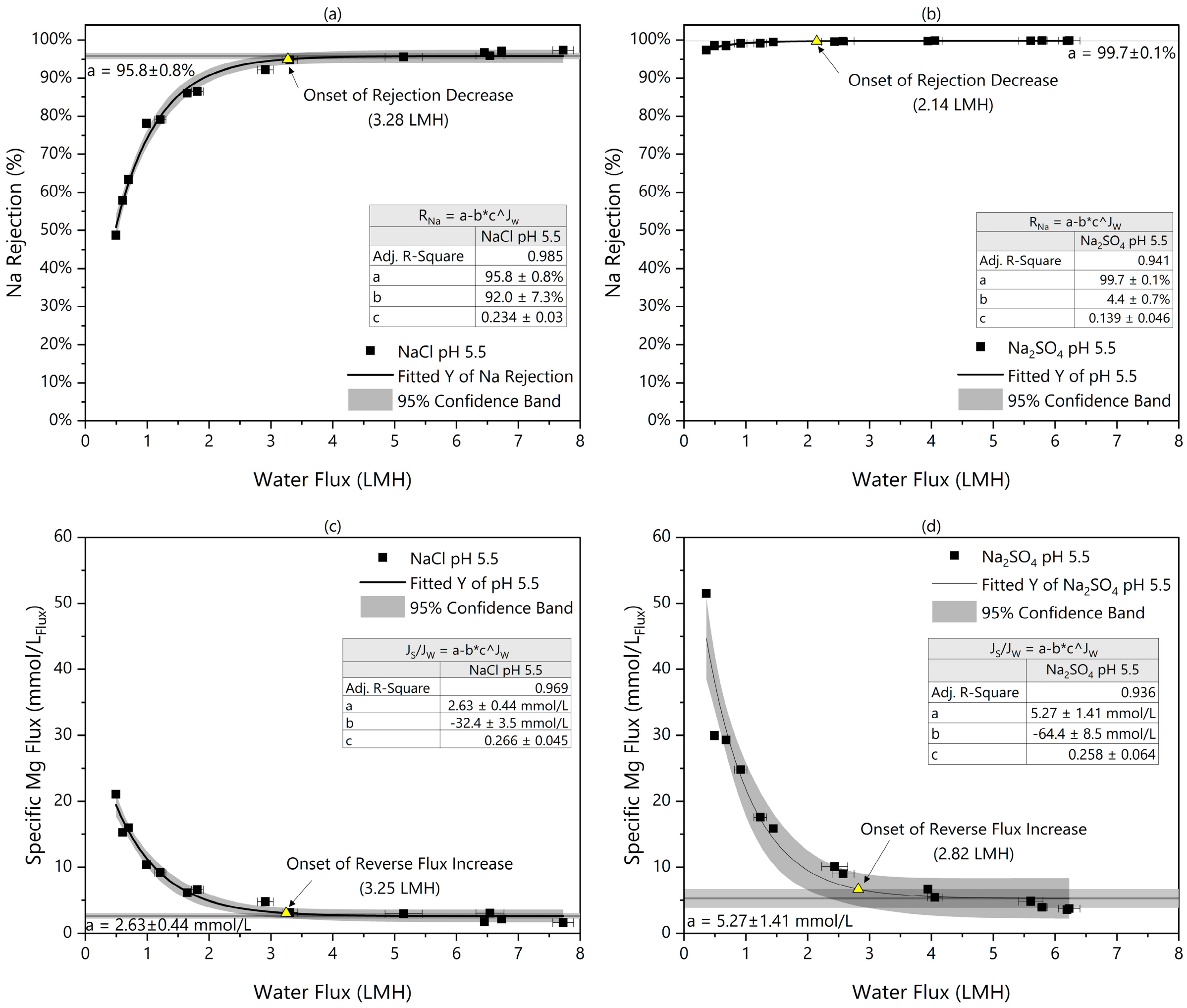

Figure S1. Examples of determining the minimum water flux for Na Rejection and Specific Mg Flux for the pH 5.5 conditions with (a,c) NaCl and (b,d) Na_2SO_4.

S Rejection at Varied pH

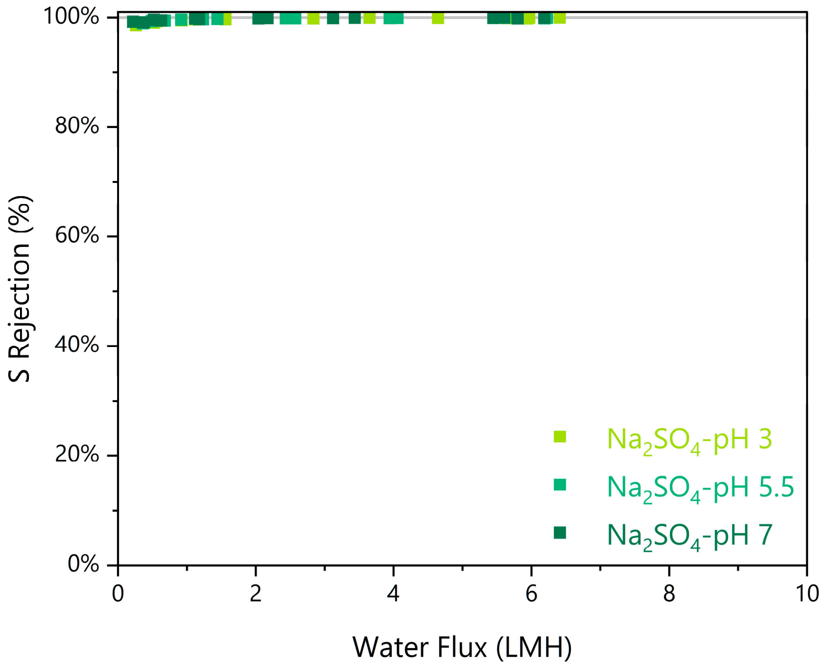

Figure S2. The S rejection plotted against the water flux for varied initial pH with Na_2SO_4 as the feed. Duplicates are shown as individual datapoints, and the error bars indicate uncertainty in the water flux measurement between the 2 balances.

Sample Experiment Time Series Data

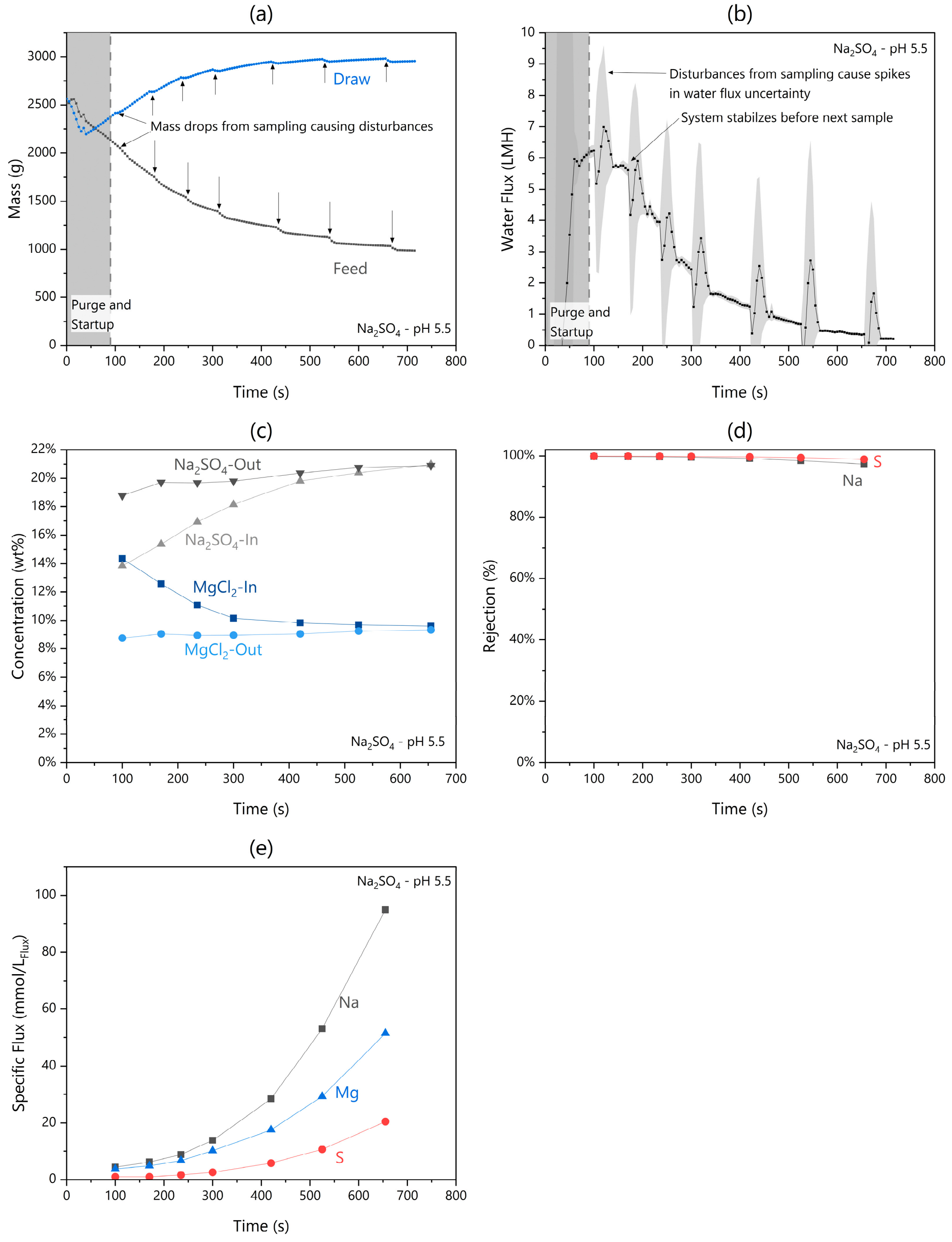

Figure S3. Sample time series data for (a) masses measured by the balances, (b) water flux (the grey shaded spikes are the water flux uncertainty caused by sampling), (c) concentrations, (d) rejection of Na and S, and (e) specific fluxes of Na, Mg, and S.
